# Fabrication of Sol Gel Solution-Based Zinc–Tin Oxide/Carbon Nanotube Hybrid Thin-Film for Thin-Film Transistors

**DOI:** 10.3390/mi16040411

**Published:** 2025-03-30

**Authors:** Yong-Jae Kim, Woon-Seop Choi

**Affiliations:** Department of Semiconductor Engineering, Hoseo University, Asan 31499, Republic of Korea

**Keywords:** oxide TFT, sol–gel, solution-processed, single-walled carbon nanotube

## Abstract

Solution-processed oxide thin-film transistors (TFTs) can lead to a significant cost-effective process and suitable for large-scale fabrication. However, they often face limitations, such as lower field-effect mobility, the use of indium which is toxic and rare, and degradation compared to vacuum-based technologies. The single-walled carbon nanotubes (SWNTs) were incorporated with zinc–tin oxide (ZTO) precursor solution without dispersants for the device’s active layer. Sol–gel solution-based ZTO/single-wall carbon nanotube (ZTO/SWNT) (TFTs) with various SWNT concentrations were fabricated to improve the performance of ZTO TFTs. ZTO TFTs containing SWNTs exhibited better electrical performance than those without SWNTs. Among the samples, the ZTO TFT with an SWNT concentration of 0.07 wt.% showed a field-effect mobility (μsat) of 13.12 cm^2^/Vs (increased by a factor of 3) and an I_on_/I_off_ current ratio of 7.66 × 10^7^ with a lower threshold voltage. SWNTs in the ZTO/SWNTs acted as carrier transfer rods, playing a crucial role in controlling the electrical performance of ZTO TFTs. The proposed fabrication of a sol–gel solution-based process is highly compatible with existing processes because it brings ZTO/SWNT hybrid TFTs closer to practical application, opening up the possibilities for next-generation electronics in flexible devices and low-cost manufacturing.

## 1. Introduction

Display technologies have diversified in form depending on the usage environment, and the demand for each pixel in displays to process multiple signals within a short period has increased because of the higher resolution. Therefore, each pixel in the display must be composed of thin-film transistors (TFTs) with excellent electrical characteristics. These transistors come in various types including amorphous silicon (a-Si) TFTs, low-temperature poly-silicon (LTPS) TFTs, and oxide TFTs. Among them, oxide TFTs are attracting attention as next-generation display driving devices because of their high charge mobility, high transparency, excellent chemical stability, and the ease of large-area fabrication compared to traditional a-Si TFTs [1].

The interest in low-cost manufacturing technologies has increased. In particular, the solution process has been actively studied as an alternative to traditional sputtering for depositing metal oxides. The solution process has several advantages over sputtering, such as eliminating the need for large chambers and high-vacuum systems and fabrication under atmospheric pressure, resulting in excellent uniformity and faster processing. The solution process can lead to significant cost-reductions [2], operate at lower temperatures [3], and enable precise tuning of material properties at low temperatures to achieve uniform nanostructures [4]. However, compared to vacuum-based technologies, solution-processed oxide TFTs have limitations such as lower field-effect mobility and rapid degradation under ambient conditions [5]. To address these challenges, many research groups have developed transparent TFTs using oxides, such as indium–zinc oxide (IZO), zinc–tin oxide (ZTO), and indium–gallium–zinc oxide (IGZO), which have demonstrated superior performance compared to conventional a-Si TFTs [6,7,8].

Indium-based materials such as indium–tin oxide (ITO), IZO, and IGZO have been widely developed for TFTs in electronic devices because of their excellent electrical and optical properties. However, issues such as the toxicity, environmental impact, high cost, and limited supply of indium indicate the need for sustainable alternative materials [9,10]. Indium residues generated during manufacturing can degrade device performance and can also increase internal stress which lead to material cracking and structural damage.

In contrast, ZTO is an indium-free oxide semiconductor with a wide bandgap [11], low cost [12], non-toxicity, and compatibility with large-scale production [13] making it a promising material for future oxide semiconductor development. ZTO has been evaluated for its gas-sensing properties and as a high-resistance buffer layer in oxide transistors for applications in thin-film solar cells and displays. Nevertheless, many challenges still remain to increase the electrical properties of solution-processed oxide TFTS while holding these advantages.

Single-walled carbon nanotubes (SWNTs) are nanomaterials comprising carbon with diameters ranging from a few nanometers to tens of nanometers and lengths of several micrometers. SWNTs demonstrate outstanding strength, elasticity, and superior electrical and thermal conductivity, significantly outperforming conventional reinforcements used in composite materials. The electronic characteristics of SWNTs are heavily influenced by their structural configuration, which determines if they display metallic or semiconducting behavior. These distinctive properties make SWNTs ideal for applications that demand efficient charge transport, including high-performance transistors and sensors [14]. The active layer of TFTs fabricated with SWNTs is much more durable and flexible than indium–tin oxide (ITO), and SWNTs function as excellent conductors in TFT devices [15]. These materials have the potential to develop devices with outstanding electrical conductivity.

Several studies have attempted to combine SWNTs with other materials to leverage the benefits of both. For example, pentacene has been used alongside SWNTs as the active channel material in TFTs [16]. SWNTs have been mixed with IZO or IGZO to enhance the electrical properties of TFTs [17,18]. These studies reported significant improvements in the mobility and electrical performance of composite TFTs incorporating SWNTs. Dispersing SWNTs using surface-active agents in solution-processed precursor solutions is always an issue to resolve. On the other hand, most of these materials rely on indium, which poses significant challenges because of its limited supply and high cost. No research was reported on the effects of combining indium-free solution-processed ZTO with SWNTs as the active channel for improving electrical properties of transistors.

Therefore, in this study, a ZTO/SWNT nanocomposite was developed using a sol–gel solution process to take advantage of the ease of large-area fabrication of ZTO and the high conductivity of SWNTs. The SWNTs were dispersed efficiently using an optimized dispersion method without a surface-active agent, resulting in the formation of a uniform and continuous thin film. This approach reduces solvent usage and promotes environmentally friendly processing, which enhances sustainability. ZTO/SWNT TFTs with excellent electrical performance were fabricated using this nanocomposite as the active layer in TFTs. This presents a novel pathway to improve the performance of oxide-based TFTs for potential electronic applications.

## 2. Experimental

Zinc acetate dihydrate and tin (II) chloride dihydrate were mixed with 2-methoxyethanol to form a stabilized sol–gel solution mixture, which served as the active layer. The concentration of the metal precursors was 0.3 M with an equal molar ratio of Zn to Sn. The solution was stirred at 50 °C for 24 h. The SWNTs (US Research Nanomaterials, Inc., Houston, TX, USA) have single-walled nanotubes > 90 wt.%, a length of 5–30 µm, and an average diameter of 1.1 nm. These were mixed with the ZTO precursor in 2-methoxyethanol and dispersed using an ultrasonic disperser at 20 °C for one hour. The SWNT concentration in the dispersion was calculated based on the mass ratio of SWNTs to the Zn and Sn precursors ranging from 0 wt.% to 0.07 wt.%.

After passing through a 5 µm filter, the precursor solution was spin-coated at 5000 rpm onto a 300 nm thermally grown silicon dioxide gate insulator on a heavily doped silicon wafer. The substrate was treated with UV/O_3_ to render its surface hydrophilic. The UV/ozone (UV/O_3_) treatment was performed using a low-pressure mercury lamp emitting at a wavelength of 254 nm. The UV intensity was set in the range of 20–30 mW/cm^2^ to induce effective photochemical activation. The samples were exposed to UV/O_3_ treatment for 30 min under ambient conditions. After spin-coating, the film was dried at 130 °C for 30 min to evaporate the solvent and annealed at 550 °C for one hour, resulting in the thickness of 60 nm. The composition of the ZTO thin-film revealed that the atomic ratio of Sn to Zn is approximately 55:45, which corresponds to a nearly 1:1 ratio. A bottom-gate, bottom-contact ZTO TFT was prepared. Aluminum was deposited on the top of the annealed ZTO layer as source and drain electrodes with 1500 µm/100 µm of width/length using a thermal evaporator with a thickness of 1100 Å and a deposition rate of 3 Å/s.

All current–voltage (I-V) characteristics were measured in a dark box at room temperature using a semiconductor parameter analyzer (Keithley 4200A-SCS, Tektronix, Beaverton, OR, USA). The surface structural properties of the thin films were examined by optical microscopy (Olympus-BX51M, Tokyo, Japan) and scanning electron microscopy (SEM, TESCAN: MAIA3, Brno, Czech Republic).

## 3. Results and Discussion

**Figure 1:**
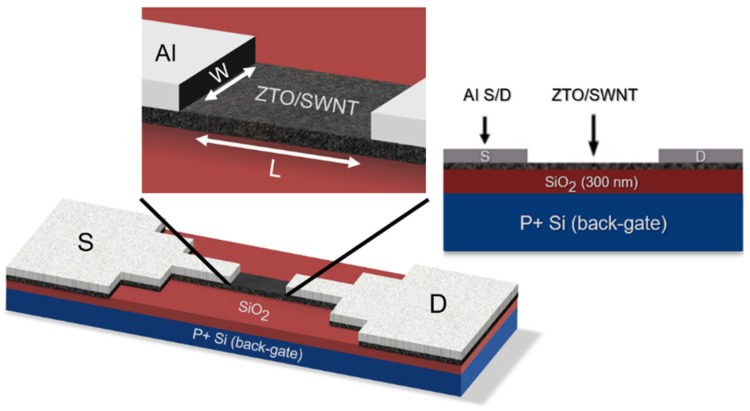
Schematic diagram of the local back-gate ZTO/SWNTs composite thin film transistor.

**Figure 2:**
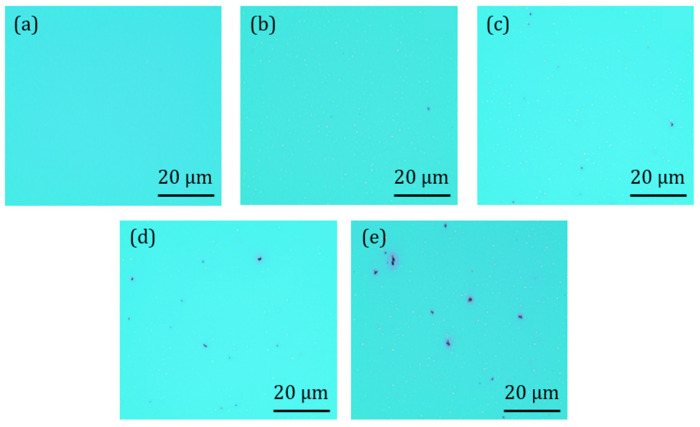
(**a**) ZTO TFT without SWNTs, (**b**) ZTO TFT with SWNTs 0.01 wt.%, (**c**) ZTO TFT with SWNTs 0.03 wt.%, (**d**) ZTO TFT with SWNTs 0.05 wt.%, and (**e**) ZTO TFT with SWNTs 0.07 wt.%.

A ZTO/SWNT thin film was formed by spin coating and followed by thermal treatment to enhance solvent evaporation and improve adhesion between ZTO and SWNT. Thin-film samples containing various SWNT concentrations (0 wt.% to 0.07 wt.%) were fabricated, as shown in Figure 1. Optical microscopy in Figure 2 showed that the amount of SWNTs on the substrate increased as the SWNT concentration increased after soft baking. These results highlight the importance of controlling the dispersion of SWNTs in the solution during preparation without any surface-active agent and emphasize the importance of the baking process in achieving high-quality data. The baking process is essential for adequately drying the ZTO/SWNT solution and stably fixing the SWNTs in the substrate. Furthermore, as the solvent evaporates, SWNTs can contact each other and aggregate if they are not well-dispersed. Clusters could form depending on the SWNT properties and content. Therefore, it is essential to check the dispersion state of the SWNTs using optical microscopy after the baking process. Ensuring a proper dispersion of SWNTs in the experiment is critical to improving the reliability of the experimental results.

**Figure 3:**
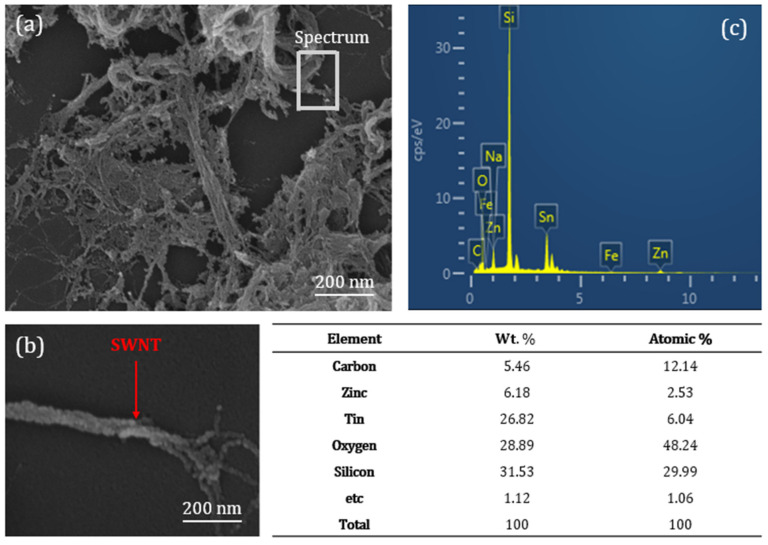
(**a**,**b**) SEM images and (**c**) EDX mapping of a ZTO/SWNTs film with a 0.05 wt.% SWNTs.

Figure 3a shows a planar image of the ZTO/SWNT transistor with a SWNT concentration of 0.07 wt.% obtained through field emission SEM (FE-SEM). The formation of percolation between ZTO and SWNTs can act as a conduction path for TFTs in Figure 3a. The formation of percolation networks can significantly affect electrical conductivity and overall device performance. To investigate the percolation effect, we varied the SWNT concentration from 0.01 wt.% to 0.1 wt.% in the precursor solution, and fabricated TFT devices accordingly. We observed that the electrical properties remained stable below a certain concentration of SWNT, but they started to rise when those concentrations increased. However, when the concentration exceeded 0.08 wt.%, the agglomeration of SWNTs increased, showing a decline in electrical characteristics. The FE-SEM image and EDX mapping in Figure 3b and c show the presence of SWNTs and the compositions of ZTO within the spin-coated ZTO/SWNT composite thin-film. The ZTO/SWNT composite thin-film prepared by spin coating exhibited a dense and continuous structure, which is expected to facilitate charge mobility between the source and drain electrodes.

**Figure 4:**
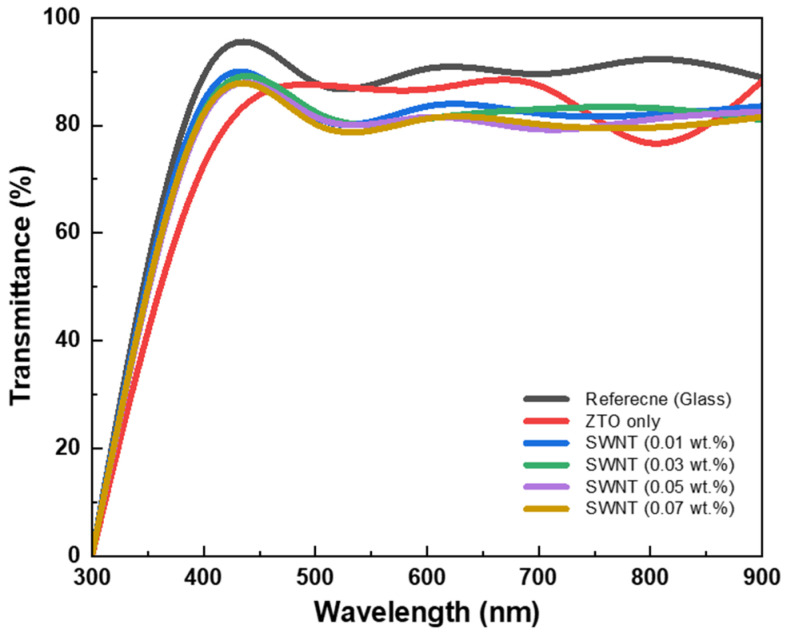
Transmittance spectrum of ZTO/SWNT nanocomposite on glass with different SWNTs.

Figure 4 presents the optical transmission spectra of ZTO/SWNT films on glass with different SWNT concentrations over the wavelength range of 300–1000 nm. The transmittance of the ZTO/SWNT thin films was lower than that of the ZTO thin films without SWNTs. On the other hand, all thin films exhibited a transmittance of over 80% in the visible region. The optical absorption coefficient (*α*) and the optical bandgap ($E_{g}$) were calculated from the transmittance data using the following equation:

$$\alpha (hv)={\left(hv-E_{g}\right)}^{1/2}$$

where h represents Planck’s constant, and v denotes the frequency of the incident photon. For direct bandgap semiconductors, the absorption coefficient typically follows a power law with an exponent of 1/2, which corresponds to the behavior of the density of states in the conduction band. The optical bandgap ($E_{g}$) increased from 3.74 eV in the absence of SWNTs to 3.82 eV when the SWNT concentration was 0.07 wt.%. This increase in bandgap was attributed to the rise in carrier concentration associated with SWNTs addition [19].

**Figure 5:**
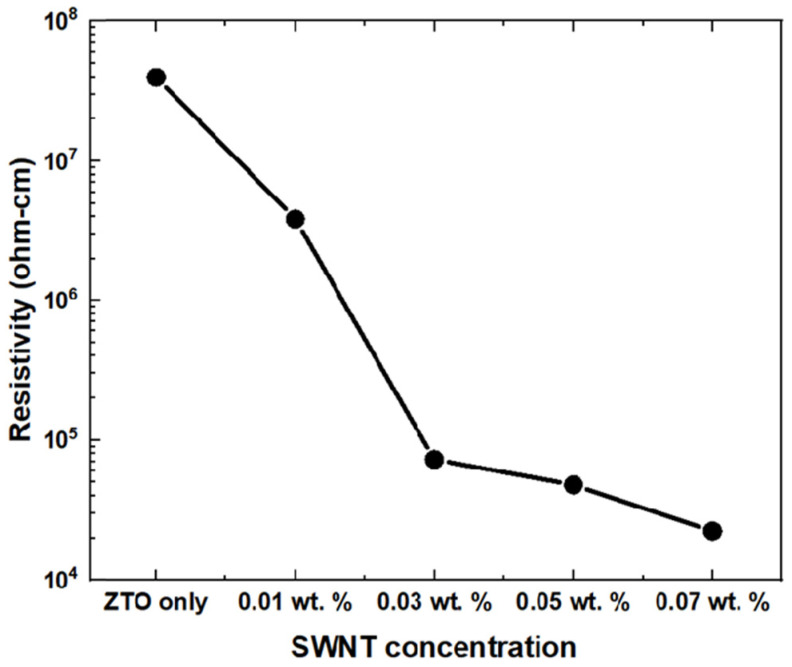
Variation in the resistivity of ZTO thin film with different SWNTs concentrations.

Figure 5 shows the resistance of the fabricated ZTO thin films as a function of the SWNT concentration (0 wt.% to 0.07 wt.%). The resistance of the ZTO thin film was approximately $7.42\;\times \;10^{7}\;\Omega \text{-cm}$ after deposition of the source and drain electrodes. Interestingly, the resistance of the ZTO/SWNT thin films decreased to $1.13\;\times \;10^{4}\;\Omega \text{-cm}$ at a SWNT concentration of 0.07 wt.%. The thin films containing SWNTs exhibited the lowest resistance. As expected, the resistance decreased as the SWNT concentration increased, suggesting that the presence of SWNTs facilitates the movement of electrons.

**Figure 6:**
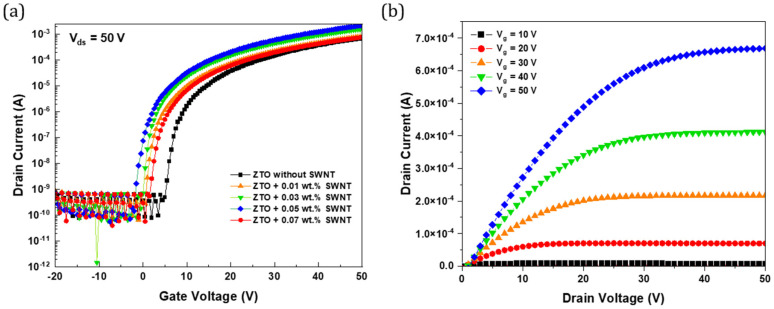
(**a**) Transfer characteristics at V_ds_ = 50 V and (**b**) output characteristics of ZTO/SWNTs composite TFTs with 0.07 wt.% SWNTs.

Figure 6a shows the transfer characteristics of ZTO/SWNT (0.07 wt.%) TFTs plotting the drain current $I_{d}$ as a function of the gate voltage $V_{g}$. The electrical parameters, including field-effect mobility, threshold voltage, I_on_/I_off_ current ratio, and subthreshold slope, were derived from a linear fit of the square root plot of $I_{d}$ versus $V_{g}$. Figure 6b presents the output characteristics of the ZTO/SWNT (0.07 wt.%) TFTs with the drain source voltage $V_{\mathrm{ds}}$ set at 50 V. The output characteristics (Figure 6b) showed no signs of confusion, indicating good contact resistance for source drain conduction. The output curves measured over a $V_{d}$ range from −20 V to 50 V exhibit a clear pinch-off and solid saturation, suggesting that the device with SWNTs follows the standard field-effect transistor theory.

The performance of ZTO TFTs with different SWNT concentrations ranging from 0 wt.% to 0.07 wt.% was compared, as shown in Table 1. The field-effect mobility (μ_sat_) increased from approximately $4.61\;{\mathrm{cm}}^{2}/V\cdot s$ for the ZTO TFT without SWNTs to approximately $13.12\;{\mathrm{cm}}^{2}/V\cdot s$ for the TFT with 0.07 wt.% SWNTs. The I_on_/I_off_ current ratio also increased significantly from approximately $2.61\;\times {\;10}^{4}$ for the ZTO TFT without SWNTs to approximately $7.66\;\times {\;10}^{7}$ for the TFT with 0.07 wt.% SWNTs. The subthreshold slope decreased from 1.91 V/decade for the ZTO TFT to 1.32 V/decade for the TFT with 0.07 wt.% SWNTs.

**Figure 7:**
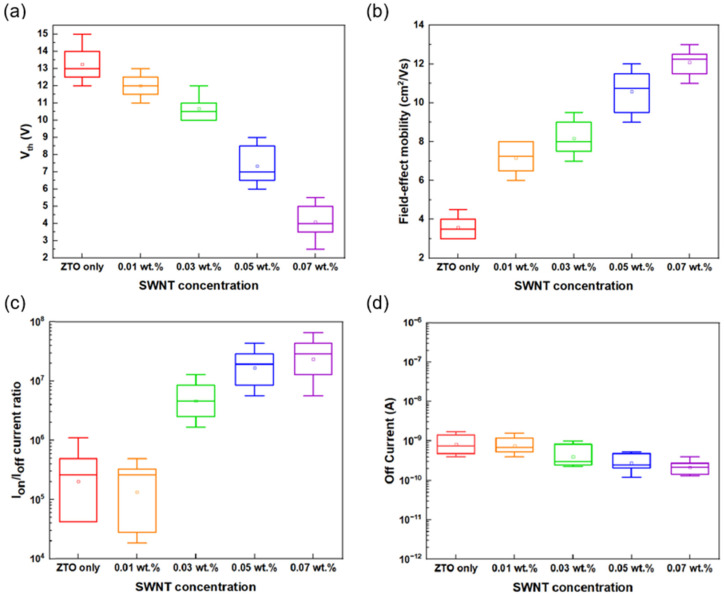
(**a**) V_th_ vs. SWNTs concentration. (**b**) Field-effect mobility vs. SWNTs concentration. (**c**) I_on_/I_off_ current ratio vs. SWNTs concentration. (**d**) Off current vs. SWNTs concentration in ZTO/SWNTs nanocomposite TFTs.

The threshold voltage (V_th_) of the TFT with 0.07 wt.% SWNTs was measured at 2.17 V. The threshold voltage (V_th_) of the TFT was also influenced by the SWNT concentration in the ZTO/SWNT TFTs. The V_th_ of the device decreases as the SWNT concentration in the ZTO/SWNT composite TFT increased, as shown in Figure 7a. The average V_th_ of the devices improved from 13.91 V to 2.17 V with the inclusion of SWNTs. Hence, the V_th_ can be tuned by adjusting the SWNT concentration. The ZTO TFT with 0.07 wt.% SWNT, which exhibited the highest electrical performance in this study, showed comparable electrical characteristics such as threshold voltage, mobility, and I_on_/I_off_ ratio when compared to the indium oxide transistor [20], and showed better properties than SnO_2_ TFTs using combustion precursors [21].

The mechanism for the improved threshold voltage (V_th_) by adding SWNTs involves metallic SWNTs on the surface of an active channel, providing some carriers needed during conduction. In addition, the SWNT concentration also affects the field-effect mobility of the ZTO/SWNT TFTs. Figure 7b shows the mobility data for TFTs with different SWNT concentrations, where the maximum mobility reached $13.12\;{\mathrm{cm}}^{2}/V\cdot s$ for TFT with 0.07 wt.% SWNTs. Conducting SWNTs provides fast tracks for carrier transport, allowing electrons to flow more quickly through the SWNTs than through the conventional channel. This leads to a significant improvement in carrier mobility.

The I_on_/I_off_ current ratio of the TFTs also increased as SWNT concentrations increased, as shown in Figure 7c. The highest I_on_/I_off_ current ratio was $7.66\;\times {\;10}^{7}$ for the TFT with 0.07 wt.% SWNTs. The correlation between the SWNT concentration and the high I_on_/I_off_ current ratio indicates that SWNTs act as carrier transfer rods facilitating electron transport in the channel region. Furthermore, the off current of the TFT devices varied with the SWNT concentrations in the ZTO/SWNT TFTs (Figure 7d). The off current for the ZTO TFT without SWNTs was approximately $6.46\;\times {\;10}^{-9}$, while the TFT with 0.07 wt.% SWNTs showed a decrease in off current of $3.54\;\times {\;10}^{-10}$.

The increase in the concentration of metallic SWNTs in the TFT leads to a decrease in off current. This suggests that in the off state, electrons are trapped by the SWNTs, resulting in a negligible off current [19]. The ZTO TFT with 0.07 wt.% SWNTs exhibited a lower off current compared to the device without SWNTs. On the other hand, the ZTO TFT with 0.08 wt.% SWNTs showed a significant increase in leakage current as the concentration of SWNTs increased because of the aggregated and interconnected SWNTs. Therefore, conductive pathways are formed between the source and drain electrodes, leading to a higher off current.

The large subthreshold swing of the ZTO/SWNT device is attributed to insufficient control of channel currents at low gate voltages and a high density of trap states. In particular, the presence of SWNTs induces localized charge traps, which deteriorates subthreshold performance. Previous studies said that CNTs creating charge traps [22] and reducing the interface trap density [23] in ZTO TFTs can improve the subthreshold swing. In this study, the large subthreshold swing of ZTO TFTs without SWNTs was improved through SWNT concentration optimization, UV/O_3_ surface treatment, and annealing processes to enhance the interface. As a result, the ZTO/SWNT device exhibited a smaller subthreshold swing compared to the ZTO device without SWNTs.

**Figure 8:**
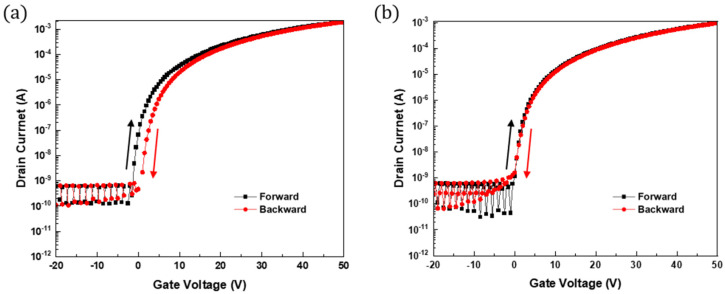
Hysteresis characteristics of a TFT. (**a**) ZTO TFT, (**b**) ZTO TFT with 0.07 wt.% SWNTs.

Clockwise hysteresis directions were observed when the gate voltage was scanned from −20 V to 50 V (Figure 8). The presence of clockwise hysteresis in the transfer curve suggests that as the voltage increases, electrons are captured by shallow energy level defects located near the interface between the gate oxide and the ZTO/SWNT active layer [24]. Figure 8a presents the hysteresis observed in ZTO as the active layer. In contrast, the ZTO/SWNT TFT with the optimal concentration of 0.07 wt.% SWNTs showed 0.74 of gate voltage shift, and almost no hysteresis, as shown in Figure 8b. The ZTO/SWNT TFT containing SWNTs exhibited reduced hysteresis compared to the ZTO TFT without SWNTs. This suggests that the minimized hysteresis can be adjusted simply by controlling the concentration of SWNTs, suggesting the possibility of operating a device with negligible hysteresis. Consequently, this material is a good candidate for preparing more reliable TFT devices.

SWNTs play a crucial role as carrier transfer rods in transistors, and the off current of TFT devices varies according to SWNT concentrations [9]. The electron mobility was enhanced as the concentration of SWNTs in the device increased, allowing them to act as efficient conductors. All devices containing SWNTs exhibited significantly improved electronic properties compared to those without SWNTs. A key challenge of the method presented in this study is the difficulty in applying high concentrations of SWNTs in the mixture. The maximum achievable concentration of SWNTs is 0.07 wt.%. Meanwhile, concentrations above 0.08 wt.% lead to the poor dispersion and aggregated state of SWNTs in ZTO films. This negatively impacts the electrical properties, causing the mixture to require the use of dispersing agents to maintain the uniform distribution of SWNTs.

**Figure 9:**
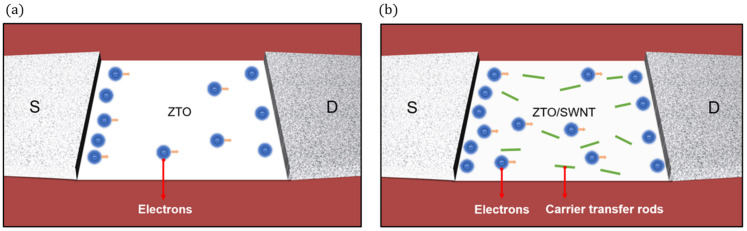
The approach to increase electrical performance in a solution-based ZTO blended with SWNTs as carrier transport rods. (**a**) ZTO TFT, (**b**) ZTO/SWNT TFT.

Charge transport mechanism in the active layer of ZTO/SWNT TFTs was proposed in Figure 9. Figure 9a represents the active layer of a ZTO device without SWNT incorporation and Figure 9b shows the active layer of a ZTO/SWNT nanocomposite device. Electrons flow through the ZTO-only channel with relatively less efficiency compared to the ZTO channel containing SWNTs. It is postulated that SWNTs act as carrier transfer rods enabling continuous electron flow and significantly enhancing the carrier mobility of the device. The percolative interaction between the ZTO matrix and SWNTs, which substantially improved carrier mobility, and the thermal treatment, which reduce charge trapping, both ultimately enhance the overall device performance. Furthermore, the uniform dispersion of SWNTs within the ZTO layer and the formation of intimate interfaces optimize the charge transport process by minimizing scattering and ensuring electrical stability.

## 4. Conclusions

This study examined how the incorporation of SWNTs in a sol–gel solution-based ZTO affects the electrical performance of the devices. The active layer for TFTs was formed in a nanocomposite using a ZTO and SWNT mixture without a dispersant. The nanocomposite thin film incorporating ZTO and SWNTs exhibited more than 80% transmittance in the visible spectrum. The changes in resistivity revealed improved electron mobility with increasing SWNT concentration. The optimal ZTO TFT with a 0.07 wt.% SWNT concentration achieved a field-effect mobility, I_on_/I_off_ current ratio, threshold voltage, and subthreshold slope of approximately $13.12\;{\mathrm{cm}}^{2}/V\cdot s$, $7.66\;\times {\;10}^{7}$, 2.17 V, and 1.32 V/decade, respectively. In conclusion, the solution-based ZTO/SWNT nanocomposite TFTs exhibited superior electrical performance to solution-based ZTO TFTs. Hence, ZTO/SWNT mixed TFTs have the potential to be high-performance devices suitable for low-cost, large-area electronics and flexible applications.

## Figures and Tables

**Table 1:** Electrical characteristics of the fabricated TFTs.

	μ_sat_ (cm^2^/V·s)	I_on/off_	I_off_	V_th_ (V)	S-Factor (V/decade)
ZTO TFT without SWNT	4.61	2.61 × 10^4^	6.46 × 10^−9^	13.91	1.91
ZTO TFT with SWNT 0.01 wt.%	6.51	1.83 × 10^5^	6.84 × 10^−9^	12.14	1.82
ZTO TFT with SWNT 0.02 wt.%	9.11	3.66 × 10^5^	7.42 × 10^−9^	10.12	1.71
ZTO TFT with SWNT 0.03 wt.%	10.82	8.14 × 10^6^	9.04 × 10^−9^	9.23	1.64
ZTO TFT with SWNT 0.04 wt.%	11.25	7.84 × 10^6^	2.46 × 10^−9^	7.11	1.52
ZTO TFT with SWNT 0.05 wt.%	11.11	2.83 × 10^6^	2.84 × 10^−10^	5.97	1.52
ZTO TFT with SWNT 0.06 wt.%	12.02	5.32 × 10^7^	3.42 × 10^−10^	4.24	1.43
ZTO TFT with SWNT 0.07 wt.%	13.12	7.66 × 10^7^	3.54 × 10^−10^	2.17	1.32

## Data Availability

The original contributions presented in the study are included in the article, further inquiries can be directed to the corresponding author.
